# Non-Contact Cross-Person Activity Recognition by Deep Metric Ensemble Learning

**DOI:** 10.3390/bioengineering11111124

**Published:** 2024-11-07

**Authors:** Chen Ye, Siyuan Xu, Zhengran He, Yue Yin, Tomoaki Ohtsuki, Guan Gui

**Affiliations:** 1 School of Communications and Information Engineering, Nanjing University of Posts and Telecommunications, Nanjing 210003, China; yechen@njupt.edu.cn (C.Y.); 1221014235@njupt.edu.cn (S.X.); 2 Department of Information and Computer Science, Keio University, Yokohama 223-8522, Japan; zhengran@ohtsuki.ics.keio.ac.jp (Z.H.); yin@ohtsuki.ics.keio.ac.jp (Y.Y.); ohtsuki@keio.jp (T.O.)

**Keywords:** cross-person activity recognition (CPAR), channel state information (CSI), generalization capability, snapshot ensemble, metric learning

## Abstract

In elderly monitoring or indoor intrusion detection, the recognition of human activity is a key task. Owing to several strengths of Wi-Fi-based devices, including their non-contact and privacy protection, these devices have been widely applied in the area of smart homes. By the deep learning technique, numerous Wi-Fi-based activity recognition methods can realize satisfied recognitions, however, these methods may fail to recognize the activities of an unknown person without the learning process. In this study, using channel state information (CSI) data, a novel cross-person activity recognition (CPAR) method is proposed by a deep learning approach with generalization capability. Combining one of the state-of-the-art deep neural networks (DNNs) used in activity recognition, i.e., attention-based bi-directional long short-term memory (ABLSTM), the snapshot ensemble is the first to be adopted to train several base-classifiers for enhancing the generalization and practicability of recognition. Second, to discriminate the extracted features, metric learning is further introduced by using the center loss, obtaining snapshot ensemble-used ABLSTM with center loss (SE-ABLSTM-C). In the experiments of CPAR, the proposed SE-ABLSTM-C method markedly improved the recognition accuracies to an application level, for seven categories of activities.

## 1. Introduction

**Figure 1:**
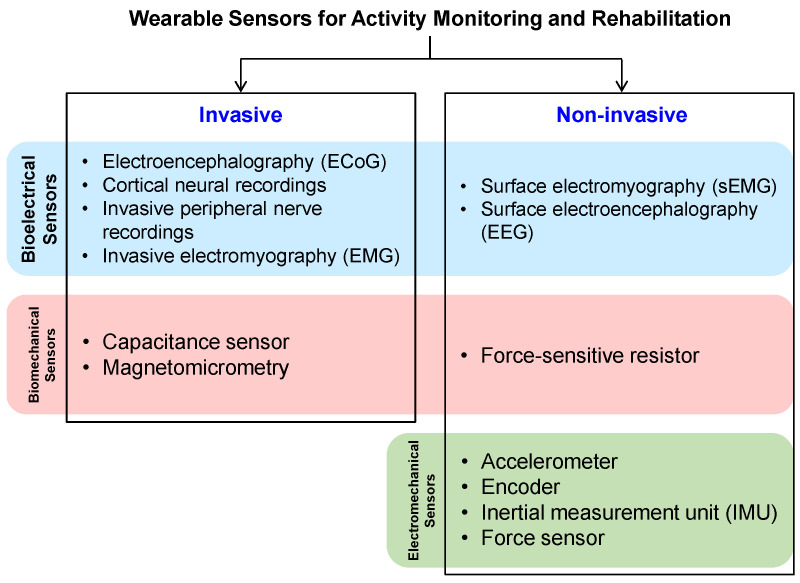
Outline of invasive and non-invasive wearable sensors for activity monitoring and rehabilitation.

Human activity recognition (HAR) is increasingly demanded in an aging society [1,2,3]. In some special periods like the recent outbreak of coronavirus disease 2019 (COVID-19), a shortage of medical resources results in the urgent need for home healthcare [4]. In the applications of HAR with walking activities [5,6,7,8], the adopted devices can be divided into two main categories, namely wearable ones [9,10,11,12,13,14,15,16,17] and non-contact ones [18,19,20,21,22,23,24,25,26,27,28,29,30,31]. Wearable devices like a wristband with an accelerometer usually can precisely identify a user’s static or motion statuses for rehabilitation and so forth, via electromyography (EMG) [15], electroencephalography (EEG) [17], and inertial measurement unit (IMU) [13], etc. Based on whether detecting the central nervous system (CNS), the invasive and non-invasive wearable sensors for activity monitoring and rehabilitation are outlined in Figure 1, referring to [10]. The invasive sensors can be divided into bioelectrical ones (e.g., electroencephalography) and biomechanical ones (e.g., capacitance sensors), besides, the non-invasive sensors further include the electromechanical ones (e.g., accelerometer). However, wearable devices may cause discomfort and skin inflammation due to direct contact with the skin [9].

In contrast, non-contact devices mainly include camera and infrared sensors based on vision [18,19,20,21], and Wi-Fi and lidar based on radio frequency (RF) signals [22,23,24,25,26,27,28,29,30,31]. In particular, over camera or infrared sensor, the use of Wi-Fi has four main strengths [22,23,24,25,26,27,28,29,30]: (1) It can penetrate non-metals like clothing and quilts; (2) It is insensitive to light and temperature; (3) It avoids the invasion of privacy; (4) Its price is uncostly. Owing to the potential of Wi-Fi-based devices for HAR, they have been adopted in numerous practical applications, e.g., home monitoring [24] and indoor intrusion detection [22].

Wi-Fi-based HAR is usually conducted by received signal strength indicator (RSSI) and channel state information (CSI) [32,33,34]. Unlike RSSI which only measures the power in a received signal, CSI can describe both the amplitude and phase of a received signal from subcarriers, which has been used in most existing HAR studies [22,23,24,25,26,27,28,29,30]. Including the transmission of Wi-Fi signals, the RF signals from a transmitter usually undergo multiple paths to reach a receiver, when the CSI is typically sensitive to the ambient environment. That is, the surrounding obstacles and human motion may reflect or interfere with the signal propagation, resulting in the variation of CSI [32,33,34]. Including our dataset, the HAR-aimed CSI datasets collected by specific tools [35], will be described in Section 2.1.

### 1.1. Conventional Wi-Fi-Based HAR Methods

HAR can be converted to a binary or multi-class classification task. Therein, the common machine learning approaches like support vector machine (SVM) [36] and random forest [37] require artificial experience for feature selection, in contrast, deep learning (DL) is a more powerful technique that can automatically extract semantic features [38,39,40,41]. Specifically, using CSI data, some DL approaches have achieved satisfactory performance in many studies on HAR [25,42,43,44,45]. Owing to the use of a convolution kernel, the convolutional neural network (CNN) usually can extract the important features of interested data, which was used to train CSI data for HAR in a transfer learning fashion as in [42]. In [28], the CSI data were converted to red-green-blue (RGB) images as the input of a two-dimensional CNN, for exploring the signal patterns of activities. Furthermore, CNN-based activity recognition was respectively studied by combining few-shot learning in [25], and multi-task learning in [43]. Considering the sequential characteristics of human activities, the recurrent neural network (RNN)-based [46] long short-term memory (LSTM) with an advanced memory capacity [47,48,49], has been introduced to handle CSI in some HAR methods [24,44,45]. In [24,44], the CSI was regarded as temporal sequences for forecasting different activities, by LSTM-included networks. Moreover, in [45], beyond only handling the past information of input samples by the standard LSTM, a bi-directional LSTM (BLSTM) also handling their future information [50] was introduced for further extracting the features of CSI. In addition, an attention mechanism that can learn the importance of features and time steps was further combined with BLSTM to present the attention-based BLSTM (ABLSTM) approach [45], obtaining improved performance.

In addition, in [26], the HAR was conducted under the scenarios across different environments and categories of activity using CSI data. In [51], a CNN-based ensemble learning strategy with heterogeneous classifiers was presented for HAR, under the same conditions of the training phase. To the best of our knowledge, most conventional Wi-Fi-based HAR methods focus on recognizing the activities of a person who has been seen in the training phase, without considering the differences in height, weight, and activity pattern, between the seen persons and unknown ones in the recognition phase, which may lack a generalization ability across different subjects [52,53,54]. In particular, the activity patterns usually vary from person to person, and the corresponding samples in HAR do not follow the same data distribution, which will hinder the generalization of classifiers. Including Wi-Fi, conventional sensor-based cross-person activity recognition (CPAR) methods via transfer learning or domain adaptation typically require additional knowledge from unknown persons during the training, such as labeled or unlabeled samples, which may be difficult in many practices [53,54]. On the other hand, some domain generalization (DG) methods with no need for target domain data on Wi-Fi-based gesture recognition were presented, e.g., WiSR in [55] and WiSGP in [56]. Note that these gesture recognition-towards DG methods are probably unable to be applied directly in CPAR, considering the differences in body size and activity pattern.

### 1.2. Our Proposal

Combining the snapshot ensemble and metric learning, this study develops a new generalized method aiming at CPAR using CSI data, i.e., to recognize the activities of unknown persons, which does not need knowledge from unknown persons in the training phase. Given the dominant performance of the ABLSTM classification network [45] on the HAR to the seen persons in the training phase, the snapshot ensemble fashion [57] is the first adopted in the ABLSTM network for obtaining SE-ABLSTM, to improve the generalization capability of recognition. Using the cosine annealing learning rate (LR), the snapshot ensemble can obtain multiple models with converged local minima of loss in one training process. To further improve the recognition generalization, we introduce metric learning to extract discriminative features with compact intra-category distance into the base-classifiers via the high-powered center loss function [58,59], by proposing the snapshot ensemble-used ABLSTM with center loss (SE-ABLSTM-C). In the activity recognition task of unknown persons, outperforming the state-of-the-art HAR method of ABLSTM [45], for each subject, the proposed SE-ABLSTM-C method improved the average accuracy of seven activities consisting of waving, clapping, walking, lying down, sitting down, falling, and picking up, approximately 3–14%. The improved generalization of our proposal to unknown persons, will be helpful for flexible health monitoring with the aid of mobile devices like robots [21].

The early results of SE-ABLSTM have been presented in our previous work as a conference version [60]. This paper further contrastively describes the use of snapshot ensemble by SE-ABLSTM, and expands the SE-ABLSTM to combine metric learning for obtaining a new SE-ABLSTM-C method. In addition, the research problem statement, the more comprehensive experiments with added subjects, and the limitation of our proposal are respectively supplemented. The code and download link of our dataset are available at https://github.com/NJUPT-Sivan/Cross-person-HAR (4 November 2024). The two main contributions of this study are summarized as follows:

1. We used the Espressif 32 (ESP32) CSI tool [34] to collect and open a CSI dataset on the mentioned seven activities in an indoor environment.
2. A new SE-ABLSTM-C method is proposed to improve the generalization ability of CPAR, obtaining higher accuracies than those of conventional HAR methods.

The organization of this paper is as follows. Section 2 presents the CSI dataset collection, and the problem and treatment by ensemble learning; Section 3 describes the framework and each part of our proposed CPAR method; Section 4 states the experiments and limitations; finally, Section 5 concludes this study.

## 2. Preliminaries

As the preliminary work, the CSI dataset collection, and the problem and treatment for CPAR, are respectively stated.

### 2.1. Dataset Collection

Over those wearable phone-based accelerometer datasets, such as WISDM (Wireless Sensor Data Mining) [61], UCI-HAR [62], and mHealth (mobile Health) [63], CSI datasets enable the non-contact sensing of human activities by the Wi-Fi technique. Reviewing the HAR-aimed CSI datasets in typical existing studies [23,24,25,26,27,28] listed in Table 1, which were usually collected on different activities from multiple subjects by several rounds in an indoor environment, with 200–1100 data. Therein, some studies [23,24,25,26] adopted the Intel 5300 Network Interface Card and Atheros for collecting CSI data, but, the 5300 CSI tool has a relatively high price and complex configuration requirement [35]. A part of CSI datasets have been public, such as those in [24,28]. In our dataset, named A, B, C, D, and E, 5 male subjects with heights of 165–175 cm and weights of 50–68 kg were respectively selected to repeat the 20 rounds of 7 activities, obtaining a total of 700 data whose scale is medium among all the mentioned CSI datasets. As a prior study on CPAR, our medium-scale dataset will be used in the experiments in Section 4, and the scale and diversity of the dataset including subjects and categories of activity, can be adjusted or extended based on actual needs. In addition, the basic information of each subject in our dataset is summarized in Table 1.

**Figure 2:**
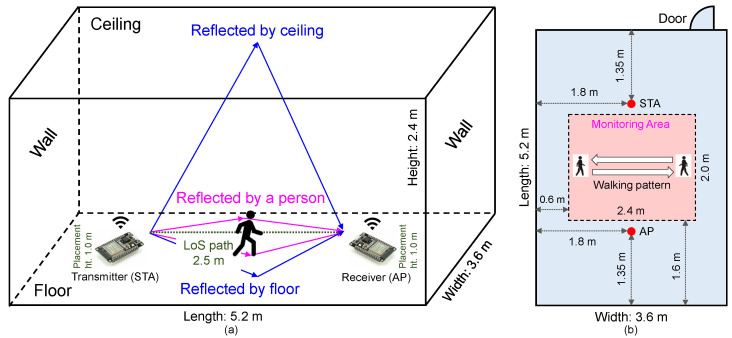
(**a**) An illustration of our CSI data collection for HAR in an indoor environment. (**b**) The layout of experiment room with the pattern of walking.

**Figure 3:**
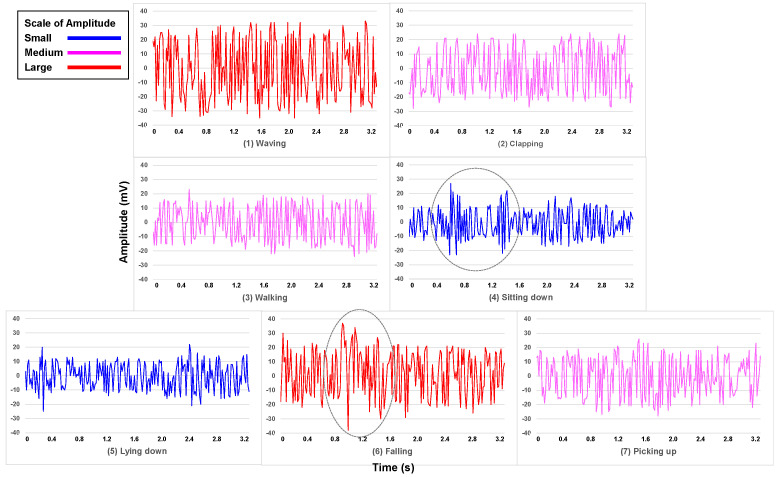
An example of raw CSI data for different activities.

Referring to recent research in [34], we adopted two ESP32 development boards consisting of a station (STA) and an access point (AP) for collecting CSI data. As shown in Figure 2a, the STA first transmits a ping command to AP as a receiver, then AP will reply to the ping command to STA, for establishing a unique connection. In an indoor environment (length: 5.2 m, width: 3.6 m, and height: 2.4 m), due to the multi-path transmission of Wi-Fi signal between the pair of transmitter and receiver, CSI will vary by the movements of a person. The layout of the experiment room with its physical dimension is shown in Figure 2b, where only two tripods of 1 m-height were respectively laid for placing STA and AP without extra furniture, and the monitoring area of 2.4 m × 2.0 m was set in the center of the room. In addition, the pattern of walking back and forth was depicted within the monitoring area, and the other six activities were conducted around the center of the monitoring area, therein a chair of 45 cm-height was used for sitting down, a common mat for lying down and falling, and an empty bottle for picking up. Figure 3 shows an example of raw CSI data samples for the seven considered activities. The amplitude scale from sitting down and lying down is roughly within −10–10 mV; the larger scale from clapping, walking, and picking up is roughly within −20–20 mV; the largest scale from waving and falling is roughly within −30–30 mV. In addition, for sitting down and falling, the highlighted sharp change of amplitudes may correspond to the occurrences of the respective activity.

The setting of the ESP32 CSI tool is listed in Table 2. By the compiler version of the Internet of Things development framework (IDF) v4.3.2, the single antenna is configured into both the transmitter and receiver, with a sampling rate of 63 Hz. The number of CSI equals that of subcarriers, i.e., 32, and the CSI with in-phase and quadrature (IQ) data in 64 ways is deemed as the considered data in this study. In ESP32, the analog-to-digital converter (ADC) calibrates the input analog voltage to the reference voltage of 1100 mV, and determines each bit of the output digital result. In a sufficiently large room, the distance of the line of sight (LoS) between the transmitter and receiver is 2.5 m, placing at the height of 1.0 m on the desks. In comparison to the numbers of antennae and subcarriers, namely 3 and 30, in the common 5300 CSI dataset as [24], our dataset adopted the compact single antenna both in STA and AP and a close number of subcarriers, namely 32.

### 2.2. Problem and Treatment by Ensemble Learning

Since the data of the activities from the targeted unknown subjects can not be obtained before the recognition phase for CPAR, some transfer learning or domain adaption approaches that need re-training of new samples may be inapplicable [52,53]. Fortunately, ensemble learning does not require additional data except those from seen subjects in the initial training phase [64].

**Figure 4:**
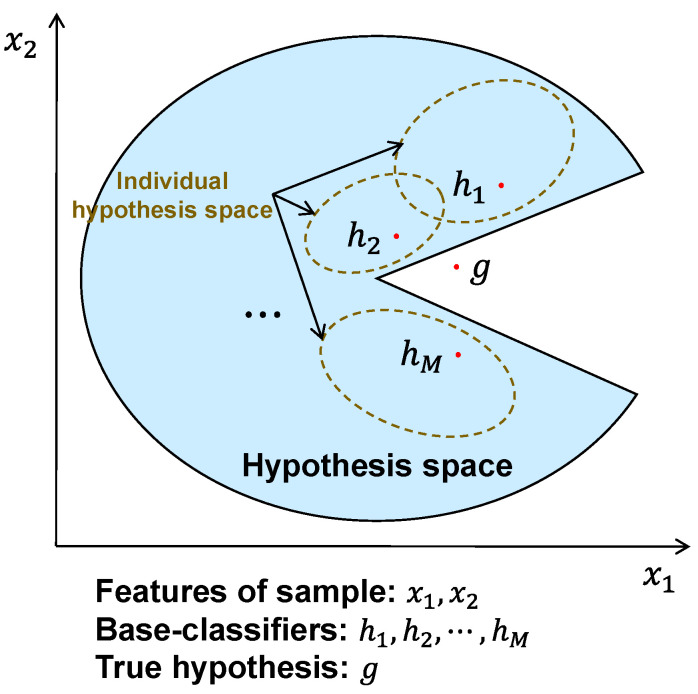
An illustration of the space expansion of individual hypotheses via ensemble, for learning a better approximation to a true hypothesis.

Figure 4 illustrates the ensemble learning-induced space expansion of individual hypotheses for approximating a true hypothesis [64]. The hypothesis space is depicted by the features of a sample $x\in R^{d},d=2$, and we assume that the ensemble contains *M* base-classifiers $h_{1},h_{2},\cdots ,h_{M}$ which are respectively trained within their corresponding individual hypothesis space. From a representation perspective, a true hypothesis *g* of the learning task may be not in the considered hypothesis space of the current learning algorithm, in this case, the use of a single classifier $h_{m},m\in \left\{1,2,\cdots ,M\right\}$ is likely to be invalid. By combining multiple base-classifiers, the space of individual hypotheses will expand, which may acquire a better approximation to the true hypothesis. In the CPAR application, the features of CSI samples from the seen persons can be deemed to construct the hypothesis space, and those from an unknown person are probably out of the constructed hypothesis space. In our proposal, the introduced ensemble strategy covering multiple individual hypothesis spaces enables a generalization improvement, helping approximate an activity category of the unknown person.

## 3. Proposed CPAR Method

The framework of our proposed CPAR method, and its two main compositions of snapshot ensemble learning and metric learning, are respectively described.

### 3.1. System Framework

**Figure 5:**
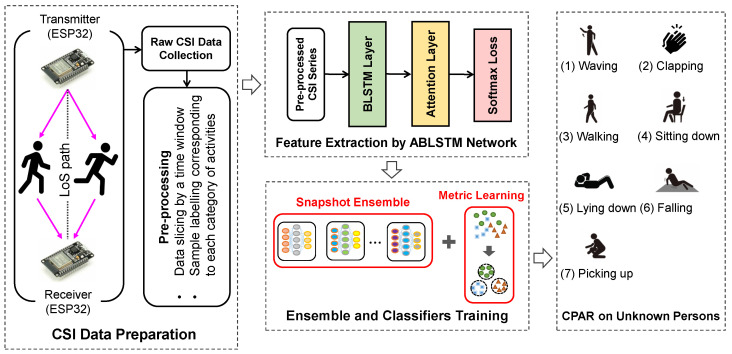
System framework of the proposed CPAR method.

The system framework of our proposed CPAR method is shown in Figure 5, consisting of four main parts: CSI data preparation, feature extraction, ensemble and classifiers training, and CPAR on unknown persons.

#### 3.1.1. CSI Data Preparation

Using the ESP32 CSI tool [34], raw CSI data was collected by the pair of transmitter and receiver on human activities. To train effective classifiers, data pre-processing is indispensable, and is composed of two steps:

- By the 3.2 s-time window with 0.8 s-forward sliding, the data is sliced into samples with 200-length. Since the observation time of each round is 16.0 s in our experiments, the number of samples becomes $17=\left(16.0-3.2\right)/0.8+1$ for each subcarrier.
- The sliced samples are labeled as 0,1,…,6 in turn, corresponding to the seven specific activities, i.e., waving, clapping, walking, lying down, sitting down, falling, and picking up.

#### 3.1.2. Feature Extraction by ABLSTM Network

**Figure 6:**
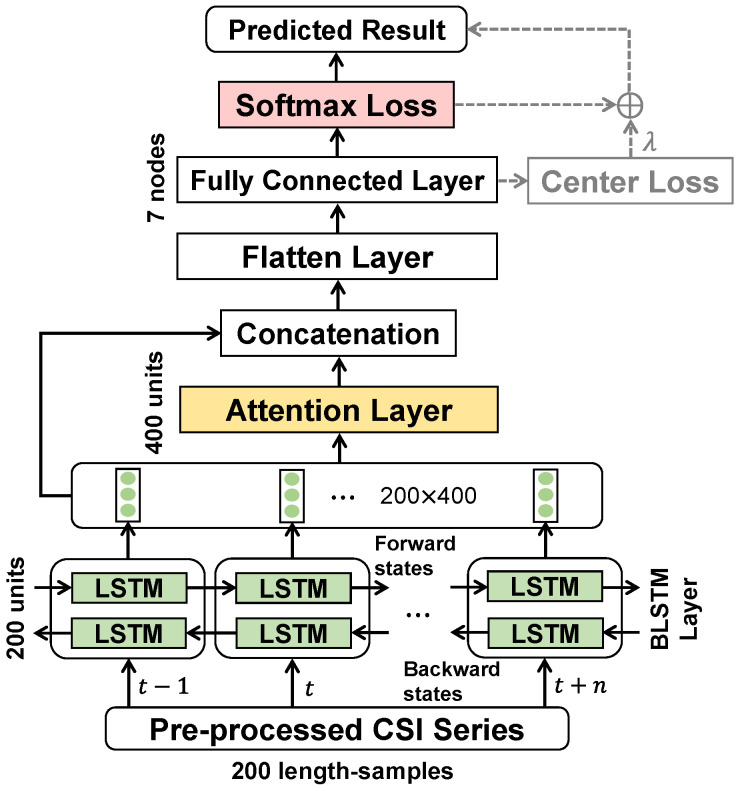
Structure of ABLSTM network. Note that center loss can be optionally combined with softmax loss (see dashed line).

In [45], the presented ABLSTM-based method has been manifested to achieve higher classification accuracy on HAR, than those of other conventional methods, such as CNN or LSTM-based methods [24,42]. Given the remarkable ability of feature extraction, the ABLSTM network is selected as the model backbone of the deep neural network (DNN) in our proposal. Reviewing the structure of the ABLSTM network [45] shown in Figure 6, first, the BLSTM layer with 200 units extracts the features of the pre-processed CSI series, namely 200 length-samples $x_{i}$, considering both the forward states (t−1→t+n) and backward states (t−1←t+n), obtaining a feature matrix with the size of 200×400. Meanwhile, the feature matrix outputted from the BLSTM layer passes into the attention layer with 400 units, which assigns weights to features and time steps by their importance, generating an attention matrix. By concatenating the feature matrix and attention matrix, a modified feature matrix sequentially passes through a flatten layer and 7 length-fully connected layers associated with the considered activities, converting into a feature vector. Finally, the converted feature vector is calculated by the softmax loss $L_{\mathrm{softmax}}$ consisting of softmax activation function $f_{\mathrm{softmax}}$ and cross-entropy (CE) loss $L_{\mathrm{CE}}$, as a predicted category vector ${\hat{y}}_{i}$. Note that the standard ABLSTM network [45] we adopted has no Transformer modules [65], which may benefit the parallel computing of sequence samples.

The softmax loss can be formulated as follows:

(1) $$L_{\mathrm{softmax}}=L_{\mathrm{CE}}\left[f_{\mathrm{softmax}}\left(z_{i}\right)\right]=-E\left\{\log\left[f_{\mathrm{softmax}}\left(z_{i}\right)\right]\right\},$$

with

(2) $$f_{\mathrm{softmax}}\left(z_{i}\right)=\frac{e^{z_{i,k}}}{\sum_{k=1}^{K}e^{z_{i,k}}},$$

where $z_{i}=\left[z_{i,1},\cdots ,z_{i,K}\right]=f_{\mathrm{FE}}\left(x_{i};W\right)$ is a non-normalized probability vector outputted from the fully connected layer, and $f_{\mathrm{FE}}$ is the mapping function from the sample space to the feature embedding (FE) space, i.e., $f_{\mathrm{FE}}:X\to Y$, which needs the substitutes of a training sample $x_{i}$ and the model weight W. $E\left[\cdot \right]$ is the operator of the expected value.

Note that the adopted structure of ABLSTM followed its original scalability only except that of the fully connected layer, and its computational load has been evaluated in [45]. The computational load of ABLSTM will approximately increase to *M* times that equals the number of cycles, in the following ensemble learning. In addition, owing to the flexibility of the ABLSTM structure, the softmax loss is combinable with other losses via a factor λ that balances the proportion of losses, such as the center loss belonging to metric learning [58,59]. More concretely, the hyper-parameters of the ABLSTM network were experimentally set in this study, which will be stated in Section 4.1.

#### 3.1.3. Ensemble and Classifiers Training

**Figure 7:**
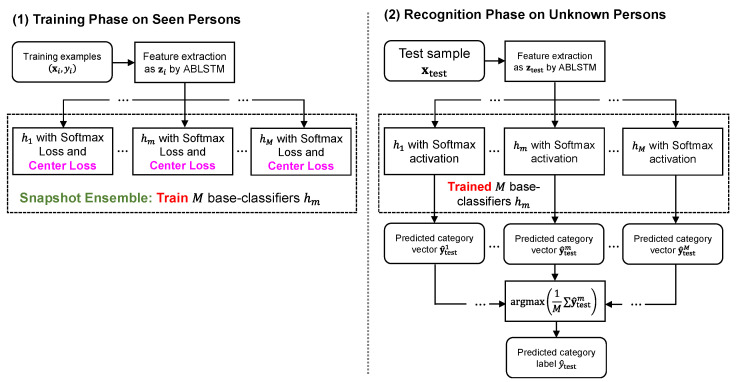
Training phase and recognition phase in the proposed CPAR method.

Figure 7 depicts the training phase and recognition phase in the proposed CPAR method. To improve the generalization ability of the ABLSTM model, an advanced ensemble strategy termed snapshot ensemble [57] is adopted, which will be described in Section 3.2. In addition, by combining the softmax loss, the center loss $L_{\mathrm{center}}$ acts on discriminative feature extraction for training high-performance classifiers [58,59], which will be described in Section 3.3.

#### 3.1.4. CPAR on Unknown Persons

Unlike the simple HAR task in which the subjects’ activities have appeared in the training phase, this study focuses on the CPAR task aiming at the activities of an unknown person. By the several trained base-classifiers, the simple averaging fashion combines their predicted category vectors for the final prediction of activity.

### 3.2. Snapshot Ensemble Learning

**Figure 8:**
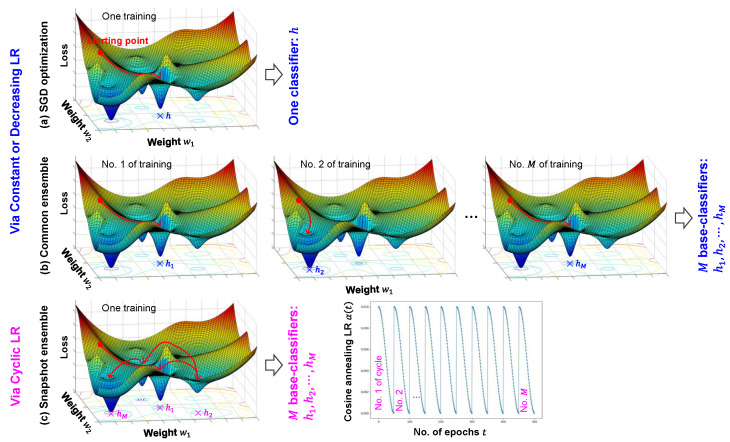
Comparison of SGD optimization, common ensemble, and snapshot ensemble. (**a**) SGD optimization via a constant or decreasing learning rate (LR). (**b**) Common ensemble via a constant or decreasing LR. (**c**) Snapshot ensemble via a cyclic LR.

Figure 8 compares the stochastic gradient descent (SGD) optimization, common ensemble, and snapshot ensemble. In Figure 8a, the SGD optimization is illustrated via a typical LR schedule that is constant or decreasing, and the model converges to a local minimum from a starting point, obtaining one classifier *h*. However, the use of a single classifier may have a poor generalization ability due to falling into a local minimum with some incorrect predictions. In contrast, ensemble learning probably improves the generalization by combining multiple classifiers with distinguishing predictions. In addition, over a single classifier, the ensemble learning may obtain a better approximation to the true hypothesis being out of the considered hypothesis space, which has been mentioned in Section 2.2. As shown in Figure 8b, unlike the only one training in the SGD optimization, the common ensemble conducts multiple training processes for obtaining one independent base-classifier in each training. More concretely, in the first training, the model converges to a local minimum from a starting point to obtain the first base-classifier $h_{1}$, similarly, *M* base-classifiers $h_{1},h_{2},\cdots ,h_{M}$ are respectively obtained in *M* training processes. However, in the different training processes of the common ensemble, the independent base-classifiers may be obtained by the models converged to the same local minimum, e.g., $h_{1}$ and $h_{M}$, resulting in some local minima not functioning in the convergence of models. As shown in Figure 8c, by the cosine annealing LR with cycles of decreasing and sharp increasing [57], the snapshot ensemble successively obtains *M* base-classifiers in only one training, whose base-models converged to different local minima with the improved generalization than that of the common ensemble.

For model training, unlike the constant or decreasing LR used in the SGD optimization or common ensemble, the schedule of cyclic cosine annealing LR in snapshot ensemble is defined as

(3) $$\alpha \left(t\right)=\frac{\alpha_{0}}{2}[\cos(\frac{\pi \;\mathrm{mod}\;\left.(t-1,\lceil T/M\rceil \right)}{\left\lceil T/M\right\rceil })+1],$$

where $\alpha_{0}$ is an initial LR, *T* is the total number of epochs, *M* is the number of cycles, and *t* is the ordinal number of epochs within each cycle. ⌈·⌉ denotes rounding up to an integer, and mod (·) denotes the modulo function that calculates the remainder of a division. In one cycle, *t* increases from 1 to [*T*/*M*], resulting in a decreasing LR α(*t*) from its maximum $\alpha_{0}$, and the model is saved until the LR decreases to a minimum. At the beginning of the next cycle, α(*t*) sharply recovers to the maximum $\alpha_{0}$, and repeats the decreasing process. In this way, *M* classifiers are successively saved in *M* cycles, during one training process. In our experiments, $\alpha_{0}$, *T*, and *M*, are empirically set at 0.01, 500, and 10, respectively. Correspondingly, the number of epochs within each cycle, [*T*/*M*], equals 50. Based on the ABLSTM only with softmax loss [45], the approach using snapshot ensemble is given in Algorithm 1, which we name SE-ABLSTM in this study.

**Algorithm 1:** SE-ABLSTM: Snapshot Ensemble-used ABLSTM  **Input:** Training dataset $D_{\mathrm{train}}=\left\{\left(x_{i},y_{i}\right)\right\}$  **Output:** Base-classifiers $h_{1},h_{2},\cdots ,h_{M}$1Construct ABLSTM [45] with softmax loss $L_{\mathrm{softmax}}$;2**Set parameters:** Initial LR $\alpha_{0}$, total number of epochs *T*, and number of cycles *M*;3**for** m=1,2,⋯,M **do**4    Obtain current model weight, i.e., **W**_*m*_(1) = **W**_cur_;
5    **for** 
t=1,2,⋯,⌈T/M⌉
 **do**
6        

$\alpha \left(t\right)=\frac{\alpha_{0}}{2}[\cos(\frac{\pi \;\mathrm{mod}\;\left.(t-1,\lceil T/M\rceil \right)}{\left\lceil T/M\right\rceil })+1];$

7        $\mathrm{Train}\;\mathrm{on}\;D_{\mathrm{train}}$;8        $\mathrm{Forward}\mathrm{propagation}:\;\mathrm{Predicted}\;\mathrm{category}\;{\hat{y}}_{i}^{m}=h_{m}\left[x_{i};W_{m}\left(t\right)\right]$;9        $\mathrm{Backward}\mathrm{propagation}:\;\mathrm{True}\;\mathrm{category}\;\mathrm{label}\;y_{i}\to \mathrm{one}\text{-}\mathrm{hot}\mathrm{vector}y_{i}$;10        $W_{m}\left(t+1\right)=W_{m}\left(t\right)-\alpha \left(t\right)\frac{\partial L_{\mathrm{softmax}}}{\partial W_{m}}$;11    
**end**
12    Obtain *h*_*m*_ with trained **W**_*m*_;13
**end**


In the recognition phase, the simple averaging for the predictions of M trained models can be formulated as follows,

(4) $$\begin{array}{c} H\left(x_{\mathrm{test}}\right)={\hat{y}}_{\mathrm{avg}}=\frac{1}{M}\sum_{m=1}^{M}h_{m}\left(x_{\mathrm{test}}\right), \end{array}$$

where $x_{\mathrm{test}}$ is a test sample, and the base-classifiers $h_{m}$ are respectively obtained by the ABLSTM in this study. Since the result of $H\left(x_{\mathrm{test}}\right)$ is in a vector form, we determine the snapshot ensemble-based final predicted category label by

(5) $$\begin{array}{c} {\hat{y}}_{\mathrm{test}}=\arg\max_{{\hat{y}}_{\mathrm{avg},k}}\left[H\left(x_{\mathrm{test}}\right)\right], \end{array}$$

where ${\hat{y}}_{\mathrm{avg},k}$ is *k*-th element of the averaged prediction ${\hat{y}}_{\mathrm{avg}}$.

### 3.3. Center Loss Belonging to Metric Learning

Although the softmax loss can deal with the separable features in a classification task, it does not dig out the discrimination characteristic of features. The separable features are usually indiscriminative, in contrast, the discriminative features are easily separable owing to their clustered distribution in the feature space. We introduce the metric learning to further improve the generalization of the proposed CPAR method, by using the center loss function that is good at recognition tasks [58,59]. The center loss aims to obtain more compact intra-category distances in the feature space, which can be formulated as follows,

(6) $$L_{\mathrm{center}}=\frac{1}{2}E\left[∥f_{\mathrm{FE}}\left(x_{i};W\right)-c_{y_{i}}{∥}_{2}^{2}\right],$$

where $c_{y_{i}}$ is the learnable center feature corresponding to $y_{i}$-th category. Combining the softmax loss in Equation (1) and the center loss in Equation (6), a hybrid metric (HM)-based loss function is composed as

(7) $$L_{\mathrm{HM}}=L_{\mathrm{softmax}}+\lambda L_{\mathrm{center}},$$

where λ acts on balancing $L_{\mathrm{softmax}}$ and $L_{\mathrm{center}}$. By replacing the softmax loss with the HM loss, the SE-ABLSTM-C approach is further obtained.

In the optimization of the center feature $c_{y_{i}}$, it should be updated when the embedded feature $z_{i}=f_{\mathrm{FE}}\left(x_{i};W\left(t\right)\right)$ changes. Considering the huge computation load for updating $c_{y_{i}}$ by the entire training samples $x_{i}$ in each epoch, a batch containing *B* training samples $x_{b}$ is usually used to calculate the feature centers of $z_{b}$ belonging to each category containing in the batch by averaging, and $c_{y_{i}}$ is updated to get close to the feature centers by

(8) $$c_{y_{i}}\left(t+1\right)=c_{y_{i}}\left(t\right)-\alpha_{\mathrm{center}}△c_{y_{i}},$$

with

(9) $$△c_{y_{i}}=\frac{\sum_{b=1}^{B}\left[\delta \left(y_{b}==y_{i}\right)\left(z_{b}-c_{y_{i}}\left(t\right)\right)\right]}{1+\sum_{b=1}^{B}\delta \left(y_{b}==y_{i}\right)},$$

where $\alpha_{\mathrm{center}}$ is the LR for the update of center loss. $\delta \left(\cdot \right)$ denotes a conditional selection function, which equals 1 if the condition is established, otherwise, it equals 0. In the case that there are no samples $x_{b}$ corresponding to $y_{i}$-th category in the current batch, namely

(10) $$y_{i}\neq y_{b},\mathrm{for}\;\forall y_{b},b=1,2,\cdots ,B,$$

$c_{y_{i}}$ remains unchanged. By combining the center loss into the SE-ABLSTM approach, the SE-ABLSTM-C approach can be obtained, which uses the HM loss $L_{\mathrm{HM}}$ for the backward propagation instead of $L_{\mathrm{softmax}}$ in the SE-ABLSTM. Correspondingly, the approach of SE-ABLSTM-C is obtained via only replacing Line 12 in Algorithm 1 by

(11) $$W_{m}\left(t+1\right)=W_{m}\left(t\right)-\alpha \left(t\right)\frac{\partial L_{\mathrm{HM}}}{\partial W_{m}}.$$

**Figure 9:**
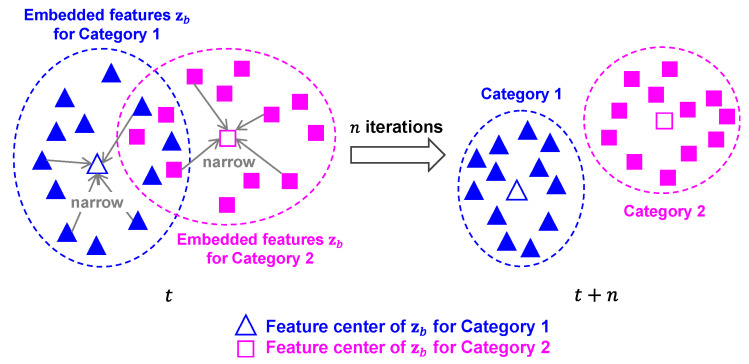
A schematic diagram of center loss.

For a better understanding, the schematic diagram of center loss for generating discriminative features is shown in Figure 9.

## 4. Experimental Results

In this section, the settings of experimental parameters and tasks, the evaluations of performance and generalization on HAR methods, and the limitation of this study, are respectively stated.

### 4.1. Parameter and Task Setting

Table 3 summarizes the parameter setting in the experiments. The information regarding the prepared CSI dataset is summarized in Table 3a. In each round of observation time of 16.0 s, the 3.2 s-time window is used to obtain a 200-length sample, with a 0.8 s-sliding window. Correspondingly, 17 samples are obtained in each round, and the total number of samples is 11,900 = 17 × 700 for our 700 CSI data (see Table 1). The hyper-parameters for model training are summarized in Table 3b. We chose TensorFlow 2.4.1 as the Python framework, the loss function combining softmax loss and center loss with an empirically selected λ=1.0, and the optimizer of Adam to train M=10 base-classifiers. The batch size and initial LR $\alpha_{0}$ are set to 128 and 0.01, respectively. In the training process, *M* cycles are set, resulting in 50 cycles in each cycle. In addition, to the comparison methods without snapshot ensemble, including CNN [42], LSTM [24], and ABLSTM [45], the LR and the number of epochs in each training are respectively $1\times 10^{-4}$ and 50.

Since the proposed CPAR method of SE-ABLSTM-C is devoted to improving generalization across different subjects rather than different environments, in the experiments, by defining the collected CSI data from the four subjects as A, B, C, and D, two kinds of tasks for HAR are respectively set as follows:

#### 4.1.1. Task I

As an auxiliary task for selecting a backbone network with high performance, the activities from a seen person are recognized, by the classifiers trained by all four subjects. The collected data are divided into one training dataset {A,B,C,D,E}, and five test datasets {A}, {B}, {C}, {D}, and {E}. By training the network model using {A,B,C,D,E}, {A}, {B}, {C}, {D}, and {E} are respectively used for HAR. The sample ratio of training, validation, and test, are 8:1:1.

#### 4.1.2. Task II

As the main task, it aims to conduct the CPAR on unknown persons, by training classifiers using the data of seen persons. The collected data are divided into four patterns consisting of training and test datasets, namely {A,B,C,D}→{E}, {A,B,C,E}→{D}, {A,B,D,E}→{C}, {A,C,D,E}→{B}, and {B,C,D,E}→{A}. For example, by training the network model using all the data in {A,B,C,D}, the data in {E} are used for CPAR. In the following experiments, we have used the abbreviation of ABCD-E instead of {A,B,C,D}→{E} for simplicity, and it is similar for other patterns.

### 4.2. Evaluation of Performance in Task I

**Figure 10:**
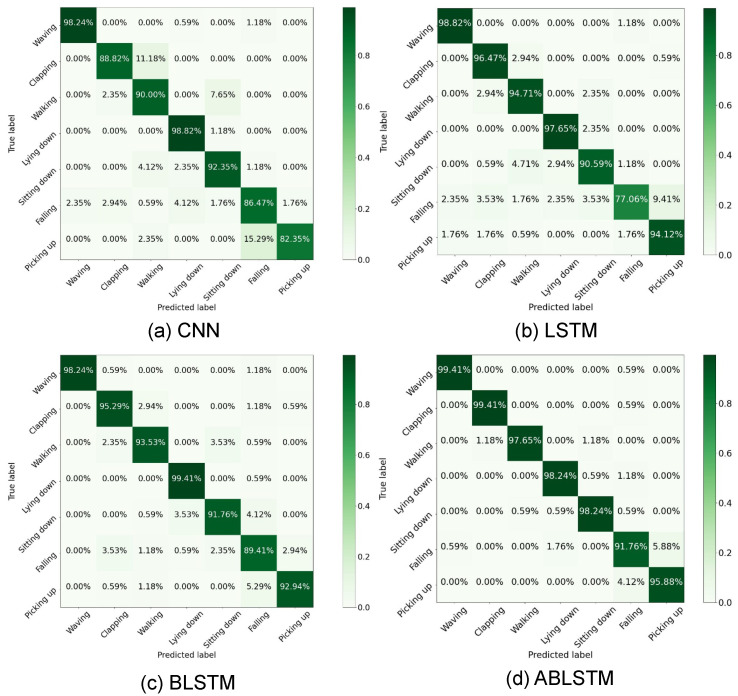
Confusion matrices by conventional HAR methods for seen persons (Task I). (**a**) CNN [42]. (**b**) LSTM [24]. (**c**) BLSTM. (**d**) ABLSTM [45].

In Task I for recognizing the activities of seen persons, the confusion matrices generated from the conventional HAR methods are shown in Figure 10, respectively. In general, all the four methods of CNN [42], LSTM [24], BLSTM, and ABLSTM [45], can relatively precisely recognize the considered seven activities of waving, clapping, walking, lying down, sitting down, falling, and picking up. Specifically in Figure 10d, most of the recognition accuracies by ABLSTM are close to 100%, such as 99.41% for both waving and clapping and 98.24% for both lying down and sitting down. Even though for the recognition results by CNN shown in Figure 10a, most accuracies are 85% at least, except for the lowest one of 82.35% for picking up. Compared with the other five activities, the accuracies for falling are lower than 90% by CNN, LSTM, or BLSTM, which may be due to the similarity of the two activities of falling and picking up.

In addition, Table 4 summarized the total average accuracies for all seven activities, respectively by the three conventional HAR methods and BLSTM. Here, the metric of average accuracy is defined by

(12) $$\mathrm{Accuracy}=\frac{TP+TN}{TP+FP+TN+FN}\times 100\%,$$

where TP and FP respectively classify the positive class as true positive and negative (false positive), and TN and FN respectively classify the negative class as true negative and positive (false negative). More concretely, better than the recognition accuracy of 91.01% by CNN, LSTM obtained 92.77% owing to its good capacity in handling sequential samples. In addition, owing to handling the samples in both forward and backward states, BLSTM further improved the total accuracy to 94.37%, and ABLSTM with an attention mechanism obtained the best 97.23%.

### 4.3. Evaluation of Generalization in Task II

To further evaluate the generalization ability of our proposal for recognizing the activities of unknown persons, the effectiveness of contrastive losses belonging to metric learning is accessed, and the ablation study and HAR methods on CPAR are respectively conducted.

#### 4.3.1. Contrastive Losses

The snapshot ensemble and the center loss belonging to metric learning, are two main compositions in the proposed SE-ABLSTM-C method. Among various contrastive losses, the triplet loss and the center loss are deemed two typical ones [58,59].

Based on ABLSTM [45], the effectiveness of triplet loss and the center loss were compared in Table 5, where we respectively named the ABLSTM with triplet loss or center loss as ABLSTM-T and ABLSTM-C. In general, combining contrastive losses with softmax loss, whether ABLSTM-T or ABLSTM-C brought about performance improvement over ABLSTM for most activities in each pattern, except that in Pattern ABCD-E. In Pattern BCDE-A as an example, relying on anchor samples, ABLSTM-T obtained higher accuracies for most activities but waving, and the average accuracy of 85.46%, over 83.57% by ABLSTM. In addition, in most patterns consisting of ABCE-D, ABDE-C, and ACDE-B, ABLSTM-C basically obtained better performances for different activities and average accuracies (74.48%, 77.46%, and 85.04%) over both ABLSTM and ABLSTM-T, owing to the getting close of features in intra-category. Given that, ABLSTM-C with the center loss is selected in the following experiments.

#### 4.3.2. Ablation Study

In our previous report [60], by using our same CSI dataset, the adopted snapshot ensemble-used ABLSTM (i.e., SE-ABLSTM) manifested its outperformance than the common ensemble-used ABLSTM on CPAR with the considered seven activities, in most patterns and total average accuracy. Given that, as another main composition in the proposed SE-ABLSTM-C method, the SE-ABLSTM is selected to verify the effectiveness of the ensemble strategy in the ablation study.

The accuracy comparison of the ablation study is shown in Table 6, for respectively verifying the effects of snapshot ensemble and center loss, by SE-ABLSTM and ABLSTM-C. Since the snapshot ensemble can improve the recognition generalization of ABLSTM by obtaining multiple base-models converged to different local minima, the SE-ABLSTM typically outperformed the ABLSTM. In Pattern ACDE-B as an example, by using cyclic cosine annealing LR, SE-ABLSTM obtained higher accuracies for all the seven activities over those by ABLSTM, and a higher average accuracy of 88.87% over 80.83% by ABLSTM. In the other three patterns (ABCD-E, ABCE-D, ABDE-C, and BCDE-A), the average accuracies by SE-ABLSTM of 81.47%, 75.83%, 78.93%, and 85.67%, were respectively higher than 81.34%, 71.63%, 76.49%, and 83.57% by ABLSTM.

Consisting with the analysis based on the results in Table 5, basically, ABLSTM-C brought about better performances for different activities and average accuracies over ABLSTM, owing to realizing the compact intra-category features. By introducing the center loss into the SE-ABLSTM approach, our proposed SE-ABLSTM-C was obtained. For the three activities of clapping, lying down, and sitting down, our proposal obtained at least three best performances to all the five patterns. Also, the proposed SE-ABLSTM-C respectively improved the average accuracies to the highest 84.37%, 78.86%, 80.74%, 89.03%, and 86.68%, in all the patterns. In addition, better than each part of the proposed SE-ABLSTM-C (i.e., ABLSTM, SE-ABLSTM, and ABLSTM-C), the performance gains of our proposal were respectively quantified by the improvements in average accuracy.

#### 4.3.3. HAR Methods on CPAR

Unlike the precise recognitions over 90% for Task I as shown in Table 4, in the five patterns (ABCD-E, ABCE-D, ABDE-C, ACDE-B, and BCDE-A) in Table 7, the average accuracies by the four conventional HAR methods, namely CNN [42], LSTM [24], ABLSTM [45], and LAGMAT (local and global alignment) that uses distance matrix for correlation representation [54], significantly degraded to 68.61–83.99% on CPAR due to the different characteristics between seen and unknown persons. Therein, as one of the state-of-the-art sensor-based CPAR methods, LAGMAT learns domain-invariant features by utilizing the local and global correlations of sensor signals only belonging to training data.

In Patterns ABDE-C and ACDE-B as examples, 82.35% and 71.76% by LSTM significantly outperformed 19.71% and 22.65% by CNN for clapping, respectively, owing to the memory capacity acting on sequential CSI data. In Pattern ABCE-D, compared with LSTM, ABLSTM further improved performances in the four activities of clapping, lying down, falling, and picking up, and the average accuracy to 85.57%. In both Patterns ABDE-C and ACDE-B, the ABLSTM as the backbone network in our proposal, obtained respectively higher average accuracies of 76.49% and 80.83%, than those of both CNN and LSTM. Better than the four conventional methods (CNN, LSTM, ABLSTM, and LAGMAT), owing to the use of snapshot ensemble and center loss, the proposed SE-ABLSTM-C obtained at least three highest accuracies for the five activities (waving, clapping, walking, lying down, sitting down) for all the five patterns, and at least one highest accuracy in the other two activities. For the average accuracies in all five patterns, the highest ones of 84.37%, 78.86%, 80.74%, 89.03%, and 86.68%, are obtained by our proposal, respectively. Furthermore, in the Student’s *t*-test [66], the *p*-values on the average accuracies for all five patterns between the proposed SE-ABLSTM-C method and the conventional CNN [42], LSTM [24], ABLSTM [45], and LAGMAT [54] methods are 0.016, 0.047, 0.038, and 0.051, respectively. All the *p*-values are less than or approximately equal to the significance level of 0.05, which suggests that our proposal markedly improved the average accuracies over the four conventional methods.

### 4.4. Limitations

**Figure 11:**
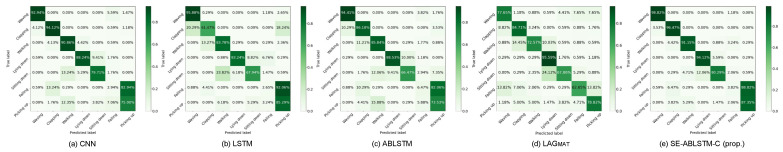
Confusion matrices by HAR methods on CPAR in Pattern ABCE-D (Task II). (**a**) CNN [42] (avg. accuracy: 74.83%). (**b**) LSTM [24] (68.61%). (**c**) ABLSTM [45] (71.63%). (**d**) LAGMAT [54] (77.01%). (**e**) SE-ABLSTM-C (78.86%).

**Figure 12:**
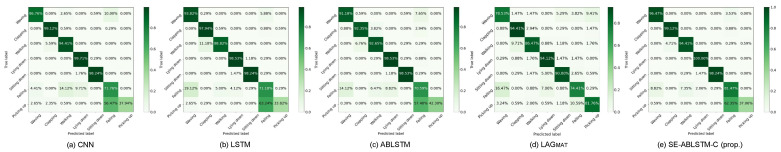
Confusion matrices by HAR methods on CPAR in Pattern BCDE-A (Task II). (**a**) CNN [42] (avg. accuracy: 83.99%). (**b**) LSTM [24] (83.29%). (**c**) ABLSTM [45] (83.57%). (**d**) LAGMAT [54] (85.67%). (**e**) SE-ABLSTM-C (86.68%).

Reviewing the relatively unsatisfied performance for falling in Task I, a similar limitation was found in CPAR in Task II. In Table 7, in Pattern ABCE-D as an example, the four conventional HAR methods (CNN [42], LSTM [24], ABLSTM [45], and LAGMAT [54]), and the proposed SE-ABLSTM-C method only obtained accuracies of 2.94–62.65% for falling, which is inapplicable to recognize the activity of falling. The confusion matrices by the mentioned five HAR methods on CPAR in Pattern ABCE-D were shown in Figure 11, where the true falling was quite easily wrongly predicted as picking up, especially for 92.06% and 88.82% respectively by LSTM and the proposed SE-ABLSTM-C. As another example in Pattern BCDE-A, the recognition accuracies of the four conventional methods and our proposal were within 33.82–81.76%, which may not satisfy a high-performance recognition of picking up. The confusion matrices by the mentioned five HAR methods on CPAR in Pattern BCDE-A were shown in Figure 12, where the true picking up was easily wrongly predicted as falling, especially for 63.24% and 62.35% respectively by LSTM and the proposed SE-ABLSTM-C.

Even though the proposed SE-ABLSTM-C method respectively obtained the two and one highest accuracies for falling and picking up, within all the five patterns (ABCD-E, ABCE-D, ABDE-C, ACDE-B, and BCDE-A), there are some low recognition accuracies, such as 3.82% for falling in Pattern ABCE-D and 37.06% for picking up in Pattern BCDE-A, as shown in Table 7. Reviewing the important finding in [29,45], the performance of the Wi-Fi-based recognition method is vulnerable to the similarity of different activities. Given that, the main reason for the low performances may be caused by the poor distinguishability of the two activities of falling and picking up, unlike the relatively good distinguishability of the other five activities. Note that the performance of LAGMAT was less affected by the similarity of falling and picking up, owing to the alleviation of distribution shifts of data among seen and unknown persons.

Fortunately, in all five patterns shown in Table 7, our proposal obtained at least 85.89% accuracies for the two obviously distinguishable activities of clapping and lying down (e.g., clapping in Pattern ACDE-B), and at least 68.82% accuracies for relatively distinguishable waving, walking, and sitting down (e.g., sitting down in Pattern ABCD-E). In the scenario that needs to ensure robust recognition of certain activities, their similar categories of activities may be excluded, such as falling, picking up, and squatting.

Recall the number of data in each dataset shown in Table 1, the scale of our dataset is to a medium extent. By combining ensemble strategy and metric learning, our proposal can be deemed a prior study on CPAR, the number of subjects may need to increase further in future exploration. Besides, since this study mainly focuses on activity recognition towards cross persons, we selected the seven typical activities of waving, clapping, walking, lying down, sitting down, falling, and picking up. The present proposed SE-ABLSTM-C method is not applicable to handle the specific classification task for falling modes, which may have different characteristics in direction or velocity.

## 5. Conclusions

Owing to the practicability of Wi-Fi-based devices, including non-contact, robustness to light and temperature, and privacy protection, we proposed a novel CPAR method named SE-ABLSTM-C for recognizing the activities of unknown persons. By adopting the snapshot ensemble, the generalization ability of our proposal on CPAR is markedly improved, based on the selected backbone network of ABLSTM. Moreover, the introduced metric learning further improves the generalization by discriminating the features from samples, over typical conventional HAR methods. In addition, our CSI dataset has been public for the development of related studies. Under the assumption of distinguishable activities, the proposed CPAR method may be applied for developing commercial products, in the healthcare of the elderly or the intrusion detection system.

In the future, the possible research directions are the CPAR with similar categories of activities, the further recognition of an activity unknown in the training phase as an open-set problem, the realization of parallel computing to sequential samples by introducing the Transformer [65], and the human gait trajectory generation combining Wi-Fi and cameras [7,21].

## Figures and Tables

**Table 1:** Dataset Description.

(a) CSI Datasets for HAR			
**Study**	**CSI Collection Tool**	**No. of Data**	**Publicity**
		**(subj. × act. × round)**	
S. Arshad, et al. [23]	5300 CSI Tool	720 (12 × 3 × 20)	No
S. Yousefi, et al. [24]	5300 CSI Tool	720 (6 × 6 × 20)	Yes
X. Ding, et al. [25]	5300 CSI Tool	200–400 (- × 4 × -)	No
Y. Zhang, et al. [26]	5300 CSI Tool	500 (1 × 10 × 50)	No
G. Forbes, et al. [27]	Raspberry Pi	1100 (1 × 11 × 100)	No
F. Moshiri, et al. [28]	Raspberry Pi	420 (3 × 7 × 20)	Yes
Our dataset	ESP32 CSI Tool	700 (5 × 7 × 20)	Yes
**(b) Basic Information of Five Subjects in Our Dataset**			
**Subject**	**Gender**	**Height (cm)**	**Weight (kg)**
A	Male	172	52
B	Male	173	60
C	Male	165	50
D	Male	175	68
E	Male	168	59

**Table 2:** ESP32 CSI Tool Setting.

Parameter	Specification
Project	ACTIVE_AP/STA
Compiler version	IDF v4.3.2
No. of antennae	1
Sampling rate	63 Hz
No. of subcarriers	32
No. of Wi-Fi channels	6
Packet rate	100 packet/s
Voltage	1100 mV
Distance of LoS path	2.5 m
Height of placement	1.0 m

**Table 3:** Experimental Parameter.

(a) CSI Dataset Preparation	
**Parameter**	**Specification**
Observation time per round	16.0 s
Time window length	3.2 s
Sliding window length	0.8 s
Sample length	200
No. of samples per round	17
Total no. of samples	11,900
**(b) Hyper-Parameter for Model Training**	
**Parameter**	**Specification**
Python framework	Tensorflow 2.4.1
Loss function	Softmax + Center
Balance factor λ	1.0
Optimizer	Adam
Batch size	128
Initial learning rate $\alpha_{0}$	0.01
No. of cycles *M*	10
No. of epochs per cycle	50

**Table 4:** Total Average Accuracy Comparison of Conventional HAR Methods for All Seven Activities of Seen Persons (Task I).

CNN [42]	LSTM [24]	BLSTM	ABLSTM [45]
91.01%	92.77%	94.37%	97.23%

**Table 5:** Accuracy Comparison on Contrastive Losses (Task II).

Approach	Waving	Clapping	Walking	Lying Down	Sitting Down	Falling	Picking Up	Avg.	Pattern
ABLSTM [45]	86.76%	92.06%	74.12%	78.24%	62.65%	89.71%	85.88%	81.34%	ABCD-E
ABLSTM-T	87.35%	91.47%	69.12%	79.41%	61.18%	90.29%	79.41%	79.75%	
ABLSTM-C	84.12%	88.53%	75.59%	81.47%	57.65%	92.06%	89.12%	81.22%	
ABLSTM [45]	94.41%	86.18%	85.84%	88.53%	66.47%	6.47%	75.53%	71.63%	ABCE-D
ABLSTM-T	97.65%	84.41%	93.81%	90.00%	69.41%	0.88%	77.06%	73.31%	
ABLSTM-C	97.35%	92.94%	85.84%	90.00%	75.79%	0.88%	78.53%	74.48%	
ABLSTM [45]	97.35%	82.06%	73.16%	99.71%	94.71%	22.12%	66.18%	76.49%	ABDE-C
ABLSTM-T	98.53%	77.35%	46.02%	100.00%	96.47%	17.40%	72.06%	72.55%	
ABLSTM-C	90.59%	81.18%	96.11%	99.71%	94.12%	26.55%	73.82%	77.46%	
ABLSTM [45]	95.29%	69.71%	81.47%	90.27%	93.24%	50.00%	85.88%	80.83%	ACDE-B
ABLSTM-T	98.53%	71.47%	93.53%	80.83%	96.47%	53.82%	80.88%	82.22%	
ABLSTM-C	97.06%	80.00%	92.35%	89.97%	95.59%	49.71%	90.59%	85.04%	
ABLSTM [45]	91.18%	92.35%	92.65%	98.53%	98.53%	70.59%	42.30%	83.57%	BCDE-A
ABLSTM-T	87.16%	98.24%	95.59%	99.41%	98.82%	74.41%	44.71%	85.46%	
ABLSTM-C	97.65%	96.47%	92.94%	98.53%	97.06%	82.06%	32.65%	85.34%	

ABLSTM-T: ABLSTM with triplet loss. ABLSTM-C: ABLSTM with center loss.

**Table 6:** Accuracy Comparison on Ablation Study (Task II).

Approach	Waving	Clapping	Walking	Lying Down	Sitting Down	Falling	Picking Up	Avg.	Avg. imp.	Pattern
ABLSTM [45]	86.76%	92.06%	74.12%	78.24%	62.65%	89.71%	85.88%	81.34%	3.03%	ABCD-E
SE-ABLSTM [60]	82.65%	96.18%	74.41%	85.29%	66.18%	76.47%	89.12%	81.47%	2.90%	
ABLSTM-C	84.12%	88.53%	75.59%	81.47%	57.65%	92.06%	89.12%	81.22%	3.15%	
SE-ABLSTM-C (prop.)	81.18%	96.47%	74.71%	87.06%	68.82%	94.41%	87.94%	84.37%	–	
ABLSTM [45]	94.41%	86.18%	85.84%	88.53%	66.47%	6.47%	75.53%	71.63%	7.23%	ABCE-D
SE-ABLSTM [60]	97.94%	94.12%	91.45%	92.94%	68.53%	2.35%	83.53%	75.83%	3.03%	
ABLSTM-C	97.35%	92.94%	85.84%	90.00%	75.79%	0.88%	78.53%	74.48%	4.38%	
SE-ABLSTM-C (prop.)	98.82%	96.47%	91.15%	94.12%	80.29%	3.82%	87.35%	78.86%	–	
ABLSTM [45]	97.35%	82.06%	73.16%	99.71%	94.71%	22.12%	66.18%	76.49%	4.25%	ABDE-C
SE-ABLSTM [60]	97.94%	85.29%	72.57%	100.00%	97.94%	28.32%	70.29%	78.93%	1.81%	
ABLSTM-C	90.59%	81.18%	76.11%	99.71%	94.12%	26.55%	73.82%	77.46%	3.28%	
SE-ABLSTM-C (prop.)	95.59%	88.53%	79.35%	100.00%	95.88%	32.45%	73.24%	80.74%	–	
ABLSTM [45]	95.29%	69.71%	81.47%	90.27%	93.24%	50.00%	85.88%	80.83%	8.20%	ACDE-B
SE-ABLSTM [60]	98.53%	85.29%	93.53%	99.12%	100.00%	54.41%	91.18%	88.87%	0.16%	
ABLSTM-C	97.06%	80.00%	92.35%	89.97%	95.59%	49.71%	90.59%	85.04%	3.99%	
SE-ABLSTM-C (prop.)	99.41%	85.59%	97.65%	97.64%	100.00%	50.88%	92.06%	89.03%	–	
ABLSTM [45]	91.18%	92.35%	92.65%	98.53%	98.53%	70.59%	42.30%	83.57%	3.11%	BCDE-A
SE-ABLSTM [60]	96.18%	95.88%	95.00%	100.00%	100.00%	75.00%	37.65%	85.67%	1.01%	
ABLSTM-C	97.65%	96.47%	92.94%	98.53%	97.06%	82.06%	32.65%	85.34%	1.34%	
SE-ABLSTM-C (prop.)	96.47%	99.12%	94.41%	100.00%	98.24%	81.47%	37.06%	86.68%	–	

SE-ABLSTM: Snapshot ensemble-used ABLSTM. ABLSTM-C: ABLSTM with center loss. SE-ABLSTM-C (prop.): Snapshot ensemble-used ABLSTM with center loss.

**Table 7:** Accuracy Comparison among HAR Methods on CPAR (Task II).

Approach	Waving	Clapping	Walking	Lying Down	Sitting Down	Falling	Picking Up	Avg.	Pattern
CNN [42]	50.59%	92.65%	67.65%	81.76%	65.29%	84.12%	65.88%	72.56%	ABCD-E
LSTM [24]	96.76%	92.65%	76.18%	82.35%	65.00%	85.29%	89.41%	83.95%	
ABLSTM [45]	86.76%	92.06%	74.12%	78.24%	62.65%	89.71%	85.88%	81.34%	
LAGMAT [54]	35.88%	91.76%	75.29%	83.24%	57.06%	73.53%	77.65%	70.63%	
SE-ABLSTM-C (prop.)	81.18%	96.47%	74.71%	87.06%	68.82%	94.41%	87.94%	84.37%	
CNN [42]	92.94%	94.12%	90.86%	88.24%	79.71%	2.94%	75.00%	74.83%	ABCE-D
LSTM [24]	95.88%	61.47%	83.78%	83.24%	67.94%	2.65%	85.29%	68.61%	
ABLSTM [45]	94.41%	86.18%	85.84%	88.53%	66.47%	6.47%	75.53%	71.63%	
LAGMAT [54]	77.65%	84.71%	72.57%	95.59%	67.06%	62.65%	78.82%	77.01%	
SE-ABLSTM-C (prop.)	98.82%	96.47%	91.15%	94.12%	80.29%	3.82%	87.35%	78.86%	
CNN [42]	96.47%	19.71%	77.88%	100.00%	96.76%	38.05%	80.59%	72.78%	ABDE-C
LSTM [24]	97.94%	82.35%	43.36%	100.00%	95.29%	9.14%	70.00%	71.15%	
ABLSTM [45]	97.35%	82.06%	73.16%	99.71%	94.71%	22.12%	66.18%	76.49%	
LAGMAT [54]	97.06%	65.59%	83.78%	95.29%	71.18%	53.69%	72.06%	76.95%	
SE-ABLSTM-C (prop.)	95.59%	88.53%	79.35%	100.00%	95.88%	32.45%	73.24%	80.74%	
CNN [42]	97.94%	22.65%	95.88%	88.79%	97.35%	66.76%	94.71%	80.58%	ACDE-B
LSTM [24]	99.41%	71.76%	95.88%	47.20%	97.35%	57.65%	77.94%	78.17%	
ABLSTM [45]	95.29%	69.71%	81.47%	90.27%	93.24%	50.00%	85.88%	80.83%	
LAGMAT [54]	91.18%	81.18%	83.53%	83.19%	79.12%	63.82%	90.59%	81.80%	
SE-ABLSTM-C (prop.)	99.41%	85.89%	97.65%	97.64%	100.00%	50.88%	92.06%	89.03%	
CNN [42]	86.76%	99.12%	94.41%	99.71%	98.24%	71.76%	37.94%	83.99%	BCDE-A
LSTM [24]	93.82%	97.94%	88.82%	98.53%	98.24%	71.18%	33.82%	83.29%	
ABLSTM [45]	91.18%	92.35%	92.65%	98.53%	98.53%	70.59%	42.30%	83.57%	
LAGMAT [54]	78.53%	94.41%	86.47%	94.12%	90.00%	74.41%	81.76%	85.67%	
SE-ABLSTM-C (prop.)	96.47%	99.12%	94.41%	100.00%	98.24%	81.47%	37.06%	86.68%	

SE-ABLSTM-C (prop.): Snapshot ensemble-used ABLSTM with center loss. In the Student’s *t*-test [66], the *p*-values on the average accuracies for all five patterns between the proposed SE-ABLSTM-C and conventional CNN [42], LSTM [24], ABLSTM [45], and LAGMAT [54] are 0.016, 0.047, 0.038, and 0.051, respectively.

## Data Availability

The code and download link of our dataset are available at https://github.com/NJUPT-Sivan/Cross-person-HAR (4 November 2024).
